# Determinants of Socioeconomic Inequalities in Traumatic Dental Injuries among Urban Indian Adolescents

**DOI:** 10.1371/journal.pone.0140860

**Published:** 2015-10-19

**Authors:** Manu Raj Mathur, Richard G. Watt, Christopher J. Millett, Priyanka Parmar, Georgios Tsakos

**Affiliations:** 1 Department of Dental Public Health, Public Health Foundation of India, Gurgaon, Haryana, India; 2 Department of Epidemiology and Public Health, University College London, London, United Kingdom; 3 Department of Primary Care and Public Health, Imperial College London, London, United Kingdom; Örebro University, SWEDEN

## Abstract

**Objectives:**

To assess socioeconomic inequalities in traumatic dental injuries (TDIs) in adolescents in New Delhi and examine the role of material, psychosocial and behavioural factors in explaining these inequalities.

**Methods:**

We conducted a cross sectional study of 1386 adolescents aged between 12–15 years residing in three diverse areas of New Delhi. A non-invasive clinical examination was used to estimate the prevalence of TDIs, and an interviewer-administered questionnaire was used to gather relevant behavioural and socio-demographic data. Multiple logistic regression models were used to assess the association between area based socioeconomic position and TDIs.

**Results:**

The overall prevalence of TDIs was 10.9%. Social inequalities in the prevalence of TDIs were observed across the adolescent population according to their area of residence. Socio-economic group differences in the prevalence of TDIs remained statistically significant after adjusting for demographic factors, material resources, social capital, social support and health affecting behaviours (OR 3.36, 95% CI 1.75–6.46 and OR 3.99, 95% CI 1.86–8.56 for adolescents from resettlement areas and urban slums respectively in comparison to middle class adolescents). Different psychosocial, material and socio-demographic variables did not attenuate the estimates for the relationship between area socioeconomic position and TDIs.

**Conclusion:**

Area of residence was a strong predictor of TDIs in adolescents with a higher prevalence in more deprived areas. Social inequalities in TDIs were not explained by psychosocial and behavioural variables. Health promoting policies aimed at improving the physical environment in which adolescents reside might be instrumental in reducing the prevalence of TDIs and associated inequalities.

## Introduction

Traumatic Dental Injuries (TDIs) have been widely recognised as a complex dental public health issue [1] which can compromise oral health and affect quality of life in children [2]. The prevalence of TDIs varies within and between countries, ranging from 5% to 58% among samples of children and adolescents from different geographical regions and in different age groups [3–7].

Socioeconomic differences in the prevalence of TDIs in adolescents are likely due to differential exposure to physical features of the neighbourhood, parental education level and employment status, and the level of social capital in the community [5,7–9]. However, associations appear to vary within and between countries, since lifestyles and levels of economic development may influence the occurrence of trauma in the population. Higher prevalence of TDIs was reported among low socioeconomic British adolescents [10], whereas studies carried out in Brazil reported higher prevalence among high socioeconomic groups [3,7,9]. Various psychosocial pathways have been suggested to explain socioeconomic inequalities in TDIs. A range of psychosocial factors, including social capital [8,11], social support [12], neighbourhood factors, as well as parents’ level of education, employment status and other socioeconomic indices have been found to be associated with TDIs [7,8]. Occurrence of TDIs has also been widely attributed to the environment which influences participation in physical activities such as sports, access to unsafe playgrounds or schools and susceptibility to road accidents and violence [13,14].

The majority of studies on TDIs in adolescents have been undertaken in developed countries and based in school settings. Very few studies have been conducted in Low and Middle Income Countries (LMICs) like India. The LMICs are undergoing a phase of rapid economic development characterised by an unplanned growth of urban areas particularly in India. Despite recent gains in reducing poverty and a rapidly expanding, upwardly mobile middle class, India remains distinguished by searing inequalities in social class and health. It is particularly problematic for an emerging and important resource for this country- it’s young. There is a clear and wide divide between those who grow up in more affluent homes in India, compared to those who grow up in less privileged contexts, in low income housing and slums. Almost 30% of the urban population of India lives in urban slums [15]. The urban poor suffer from adverse health outcomes which are generally not revealed in the commonly available statistical health records. More than 400 million children and adolescents, the most of any country in the world, resides in India [16].

None of the studies examining socioeconomic inequalities in TDIs have included extremely deprived settings such as urban slums. A small number of studies in India have assessed the prevalence of TDIs in adolescents but none has examined whether inequalities exist in the form of a social gradient or looked at the combined contribution of psychosocial (social capital and social support), behavioural and material (standard of living) factors on socioeconomic inequalities in TDIs.

This study aimed to address this gap in the literature by assessing socioeconomic inequalities in TDIs among Indian adolescents residing in three different residential areas, namely middle class (relatively affluent) and two groups of extreme social disadvantage (resettlement colonies and -even worse- slums). We further examined the role of material, psychosocial and behavioural factors in explaining these inequalities.

## Methods

**Fig 1:**
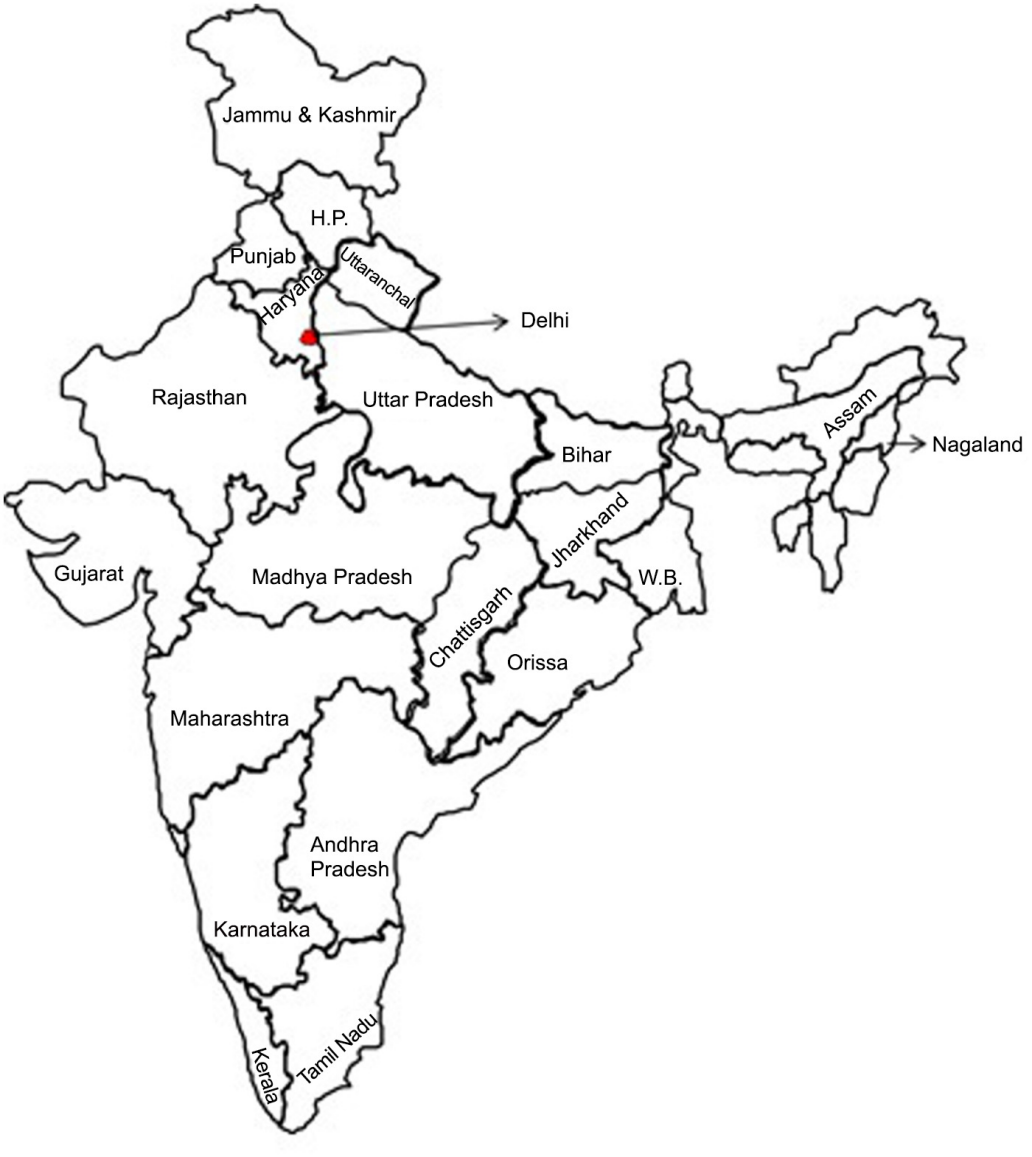
Political Map of India Showing Study Location (State of Delhi).

**Fig 2:**
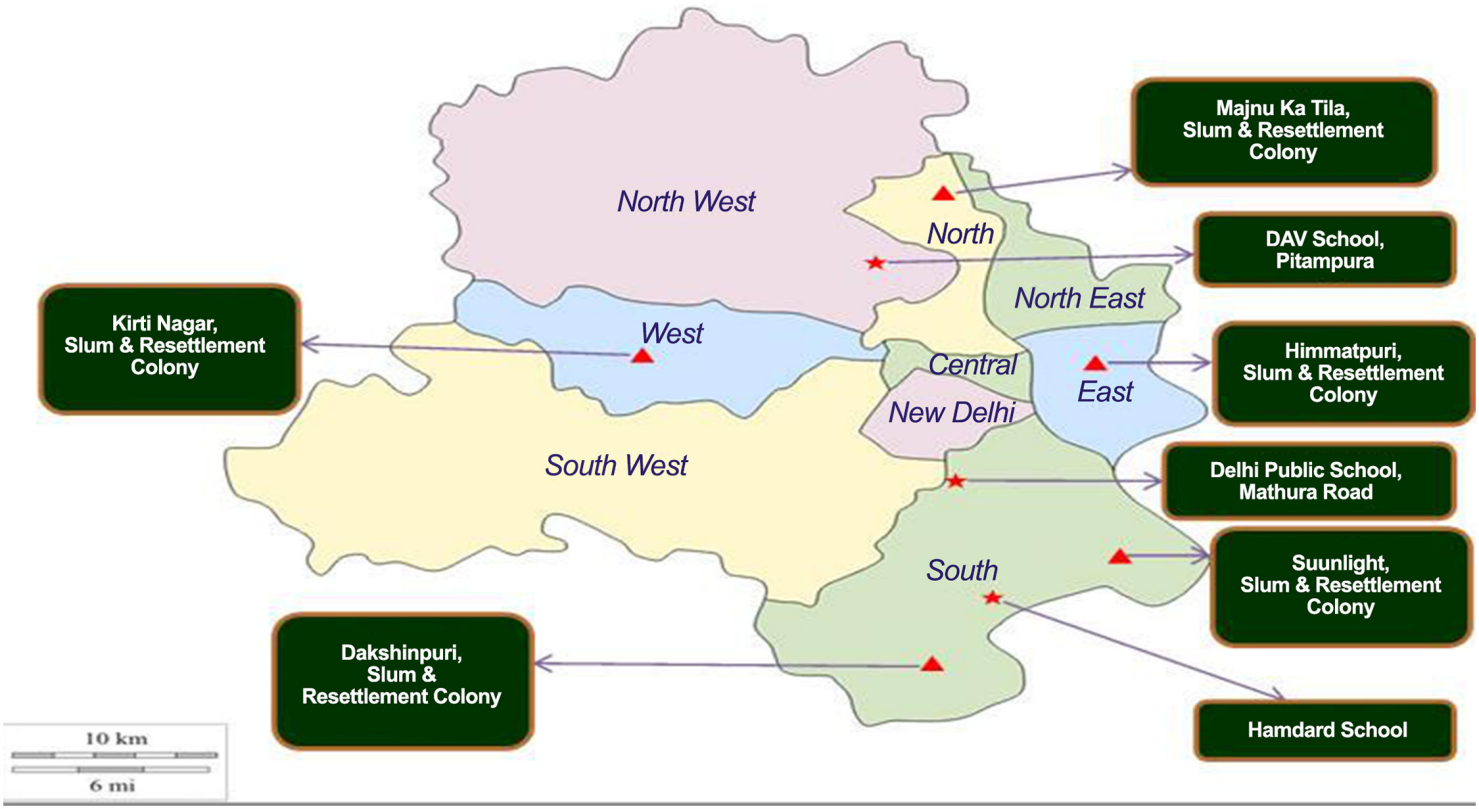
Political Map of State of New Delhi Depicting Communities and Schools Where Study was Undertaken.

A cross sectional study was carried out on adolescents aged 12–15 years living in the National Capital Territory (NCT) of Delhi. Adolescence is a very crucial phase in the life cycle of an individual [17]. It is an important phase for oral health also, as individuals get their complete permanent dentition during this period and gain independence in making personal and dietary choices [17]. The most vulnerable periods of traumatic dental injuries also occur in adolescence. We selected adolescents residing in three diverse residential areas: urban slums, resettlement colonies -which are colonies created by removing a group of households from the congested slums of city core or an encroachment in public places and are slightly better off economically than urban slums [18], and middle class neighbourhoods. Slums and resettlement colonies were recruited from a list of registered resettlement colonies and urban slums in Delhi. The inclusion criteria were a) colonies must be within a radius of 25 Km. from the research office, b) both slum and resettlement colony present together as a cluster, c) have more than 500 households in both the components of the cluster, and d) have a known non-governmental organization working in the community which is willing to participate in the research. There were 14 eligible colonies and slums. In order to assess variation in their population features, we collected demographic data from 2 blocks in each of these slums and resettlement colonies. All the slums and resettlement colonies were comparable to each other demographically (ethnicity, religion, language, number of households, population per block and school going/non-school going children per family). For the adolescents from middle class neighbourhoods, we identified private schools which have English as medium of education and charge higher fees (‘English Medium Schools’). We used multi-stage random sampling technique to select our study sample. Four slums and resettlement colonies were selected from the 14 identified colonies and four English medium private schools were randomly selected from a list of 48 eligible schools. A detailed methodology of the study has been described elsewhere [19]. The geographical map of the study areas where the study was conducted is depicted in Figs 1 and 2.

The data were collected through clinical oral examinations and interviewer-administered questionnaires. The clinical examination was used to estimate the prevalence of TDIs. TDIs were classified based on the modified version of O’Brien’s classification [20] and were recorded by two trained and calibrated dentists. Inter-examiner and intra-examiner reliability was checked by repeating the dental examinations on 70 adolescents (5% of the sample). Cohen’s unweighted Kappa coefficient was used to assess agreement. Both inter-examiner and intra-examiner agreement were above 0.86 (very high internal consistency).

An interviewer administered questionnaire was used to gather relevant demographic, behavioural and social data. The questionnaire obtained information on participant’s socioeconomic position, material resources, neighbourhood social capital, level of social support and prevalence of health related behaviours like alcohol use, diet, dental visit, oral hygiene practices, and involvement in violent activity. The questionnaire included previously used questions and scales which were further evaluated for reliability and validity in the study population during a pilot study. Material resources were assessed using India’s National Family Health Survey (NFHS) Standard of Living Index. Social capital (bridging and bonding types measuring trust, norms and reciprocity in a community) was measured by a scale developed by Gage et al (2005) [21]. This scale has been adapted from WHO Health Behaviour in School Children (HBSC) study [22]. Social Support was measured by using the Social Support Scale for Adolescents (SSAS) developed by Seidman et al (1995) [23]. The questions to assess health-related behaviours in adolescents such as diet, tobacco and alcohol use, brushing frequency, visit to a dentist, getting bullied and involvement in physical fight were also derived from the WHO HBSC survey [22].

Socioeconomic position was the primary explanatory variable in this study. This was measured by area of residence categorised into slums, resettlement colonies and middle class homes. The outcome variable was the presence of TDI. Different categories of TDIs were collapsed to create a binary aggregate variable (no dental injury vs. presence of dental injury) to calculate its prevalence.

### Statistical Analyses

Descriptive statistics were used to assess the frequency distributions of the explanatory and outcome variables. We calculated the prevalence of TDIs for adolescents according to their area of residence. Multiple logistic regression analysis was used to assess the association between area of residence and TDIs. The first model presented the unadjusted association with only the explanatory variable (area of residence) and the outcome (TDIs). In the second model, we adjusted the association between area of residence and TDIs for age, sex and religion. Different covariates were sequentially adjusted in the subsequent models in the following order; material resources, social capital, social support and health related behaviours. The final model adjusted simultaneously for all these factors in order to examine the relative influence of material resources, social capital, social support and health related behaviours in explaining the inequalities in TDIs between the three groups of adolescents. All analyses were conducted in Stata 12. Supporting information file (S1 File) contains the dataset for this study.

Ethical approval for this study was obtained from University College London Research Ethics Committee (UCL ID 2339/01) and Public Health Foundation of India, Technical Review and Institutional Ethics Committee (vide their letter number: TRC-IEC 47/10). Written and witnessed informed consent was taken both from adolescents who participated in this study and their parents/ guardians. Respondents from which any one of the consents were not obtained were dropped from the study sample.

## Results

A total of 1386 adolescents participated in the study representing a response rate of 86.6%. The mean age of the sample was 13.4 years and 53.1% were boys. 460 (33.2%) adolescents belonged to the middle classes, 462 (33.3%) were from resettlement colonies and 464 (33.5%) from urban slums. The majority of adolescents were Hindus (76.0%), followed by Muslims (20.9%) and the remaining (3.2%) belonged to other religions (Table 1).

**Table 1:** Distribution of study population based on age, sex, gender and religion (n = 1386).

Variable	Response	N	(%)
**Number of Respondents**	Middle Class	460	33.2
	Resettlement Colonies	462	33.2
	Slums	464	33.4
**Gender**	Boys	736	53.1
	Girls	650	46.9
**Religion**	Hindu	1053	76.0
	Muslim	289	20.9
	Others	44	3.2
**Age**	12 years	379	27.3
	13 years	370	26.7
	14 years	287	20.7
	15 years	350	25.2

Mean Age = 13.4 years, s.d = 1.1

**Fig 3:**
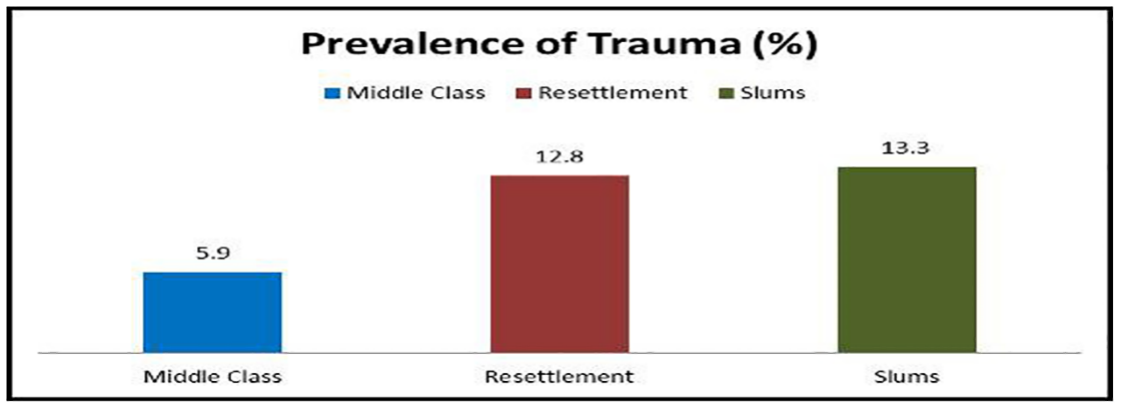
Prevalence of Traumatic Dental Injuries.

The overall prevalence of TDIs in anterior teeth was 10.9%. Lowest prevalence of trauma to anterior teeth was found in middle class adolescents (5.9%) with higher prevalence among adolescents from resettlement colonies (12.8%) and urban slums (13.3%) (p<0.0002) (Fig 3).

Boys had significantly higher prevalence of TDIs than girls (12.6% and 8.5% respectively) (Table 2).

**Table 2:** Socio-demographic distribution of Traumatic Dental Injuries (n = 1386).

Variable	Response	N	(%)
**Adolescents with TDIs**		148	10.9
**Area of Residence**			
	Middle Class	27	5.9
	Resettlement Colonies	59	12.8
	Slums	62	13.3
**Gender**			
	Boys	93	12.6
	Girls	55	8.5
**Religion**			
	Hindu	102	9.7
	Muslim	289	14.5
	Others	44	9.1
**Age**			
	12 years	39	10.3
	13 years	370	9.7
	14 years	287	14.3
	15 years	350	9.1

Table 3 presents associations between socioeconomic position of adolescents and TDIs. Compared with the middle class adolescents, the unadjusted odds of having dental trauma were 2.34 (95% CI 1.46–3.77) times higher for adolescents from resettlement colonies and 2.47 (95% CI 1.54–3.96) times higher for those living in urban slums.

**Table 3:** Association between area of residence and prevalence of dental trauma (N = 1386).

Area of Residence			
	Middle Class	Resettlement Colony	Slums
O.R (95% CI)			
Model 1	1	2.34 (1.46–3.77)***	2.47 (1.54–3.96)***
Model 2	1	2.54 (1.56–4.12)***	2.51 (1.56–4.05)***
Model 3	1	3.46 (1.83–6.55)***	3.99 (1.91–8.37)***
Model 4	1	2.51 (1.54–4.10)***	2.46 (1.51–4.03)***
Model 5	1	2.62 (1.60–4.30)***	2.69 (1.63–4.44)***
Model 6	1	2.42(1.48–3.97)***	2.56 (1.58–4.13)***
Model 7	1	3.36 (1.75–6.46)***	3.99 (1.86–8.56)***

Model 1: Unadjusted; Model 2: Adjusted for age, sex and religion; Model 3: Adjusted for age, sex and religion & material resources; Model 4: Adjusted for age, sex and religion & social capital; Model 5: Adjusted for age, sex and religion & social support; Model 6: Adjusted for age, sex and religion & health affecting behaviours (physical fight, alcohol use, dental visit and getting bullied); Model 7: Adjusted for age, sex and religion, material resources, social capital, social support & health affecting behaviours (physical fight, alcohol use, dental visit and getting bullied)

***p< 0.001

There were minor changes in the odds ratios when the relationship between area of residence and TDIs was adjusted for age, sex and religion (Table 3, model 2); social capital, social support and health affecting behaviours (Table 3, models 4, 5 and 6 respectively). However, odds ratio increased considerably when this relationship was adjusted for material resources (Table 3, model 3) and this was also the case in the final model (after adjusting for all the covariates) (Table 3, model 7). Adolescents from resettlement colonies had 3.36 (95% CI 1.75–6.46) times higher odds of having dental trauma then adolescents from middle class homes after fully adjusting for demographic factors, material resources, social capital, social support and health affecting behaviours. Similarly, adolescents from urban slums had a 3.99 times (95% CI 1.86–8.56) higher odds of having dental trauma in comparison to their counterparts from middle class homes.

## Discussion

The study showed clear social inequalities in the prevalence TDIs in adolescents residing in diverse residential areas of New Delhi. A threshold effect was observed where adolescents from middle class homes had the lowest prevalence of TDIs and adolescents from slums and resettlement colonies showed almost similar and rather higher prevalence of dental trauma. The difference in the odds of prevalence of TDIs between the three groups was statistically significant. Adjusting for neighbourhood social capital, social support and health affecting behaviours did not attenuate the existing gap for inequalities in traumatic dental injuries between the deprived groups (resettlement colonies and slums) and middle class group.

The association between area of residence and TDIs corroborates with other studies conducted on children and adolescents [11,24,25]. Jung et al (2011) in their study on South Korean adolescents showed a flattening of gradient in the prevalence of fractured teeth at the lowest two levels of self-reported socioeconomic status [24]. This concurs with the findings of our study as a threshold effect was observed in the prevalence of traumatic dental injuries with adolescents from middle/ upper middle class homes reporting the lowest and adolescents from resettlement colonies and urban slums reporting almost equal prevalence of dental trauma. In our study we used area of residence as a measure of socioeconomic position and a clinical measure of traumatic dental injuries [20]. In contrast, the study by Jung et al (2011) used self-reported socioeconomic status as a measure of SES and self-reported prevalence of fractured teeth [24]. However, the results from both the studies showed that there was a steep degree of decline in trauma levels as we moved below a certain socioeconomic position after which the gradient became less pronounced or flattened. This threshold effect separates the lowest categories of socioeconomic position with the remainder of the study population. This may be due to some innate property of the area or the physical environment in slums and resettlement colonies which leads to adolescents succumbing to more dental injuries. Pattussi et al. (2006) explored the influence of neighbourhood social capital on observed inequalities in TDIs in Brazilian boys and reported significantly lower prevalence of TDIs in neighbourhoods with higher levels of social capital [11].

We found a strong association between area of residence which was used as a measure of socio-economic position and prevalence of TDIs among adolescents. Adjusting for age, sex and religion did not explain social inequalities in TDIs between the deprived groups (resettlement colonies and slums) and middle class group. Socio-economic differences in TDIs were maintained after adjusting for social capital, social support and health related behaviours (physical fight, alcohol use, dental visit and getting bullied). This may be explained because of the specific population groups chosen for this study as two of them (urban slums and resettlement colonies) live in extreme deprivation and it is possible that the different mediating pathways may have a limited role in such extreme conditions.

Adjusting for material resources led to a higher odds ratio for the relationship between area of residence and prevalence of TDIs. Material resources in our study was measured using the Indian National Family Health Survey (NFHS) standard of living index [26]. This index was first developed in 2000 and assessed the availability of basic material things required for living by an individual. India, in the past few years has taken rapid strides towards economic development which has led to a general improvement in the standard of living. This might have resulted in some of the very basic items used in the NFHS standard of living index and the weights given to each item being not relevant anymore, thereby questioning the appropriateness of the whole scale. However, this is the most accepted measure to assess material deprivation or standard of living and is used in probably all National Surveys conducted in India. There is also a possibility that adolescents with relatively higher levels of material resources in the resettlement colonies and the slums are more prone to TDIs than those with less resources in these areas (like access to media promoting violence and access to other habit forming and intoxicating substances). Material resources in particular act as facilitator of TDIs in those settings rather than as protective factor of TDIs which could also be the case for middle class adolescents.

The social capital questionnaire which we used in our study measured the trust and norms of reciprocity in the society but did not measure the level of social or community participation (such as membership of any voluntary organization, cricket club, working for a NGO, political activism). Different studies have shown that the membership of voluntary organizations like churches, sports group and hobby groups are very strongly and inversely linked with mortality and various diseases [27,28]. Social capital might act by improving access to services and amenities. Sampson (1997) argued that neighbourhoods with high social capital can work together and fight economic hardships like budget cuts [29]. High social capital groups can easily form organizations or groups to advocate for better services like transport, health centres and recreation clubs [30,31]. Social isolation at a community level, which is very common in deprived areas like slums where there are high migration and new settlement rates, is also an important dimension which was not captured by the social capital scale used in our study. Various studies have linked it to poor health outcomes [32,33] and there is a possibility that inclusion of social isolation variable might have affected our results.

Kahn and Antonuci (1980) stated that social support is transactional in nature, which involves both receiving as well as giving in the subset of a person’s relationships [34]. George (1986) further strengthened this argument by stating that “this exchange or transaction occurs in a normative framework where behaviours are guided by norms of solidarity and independence” [35]. Social support also takes place in a life course context and not merely on a day to day need basis within the context of social networks [36]. There are scales which have measured the social interaction aspect of social support [37,38]. Our scale of social support measured mainly the received support or support available to an adolescent and did not assess the aspect of ‘giving’ support to others which might have influenced the results. However, the scale used by us was specifically developed for adolescents and was comprehensive as it assessed different dimensions of social support.

Various authors have suggested area based measures to have an important role in determining health and explaining inequalities in health [39–41]. Pickett and Pearl (2001) systematically reviewed the literature on the effect of neighbourhood as a socioeconomic predictor of health outcomes and concluded that there is sufficient evidence to show that area of residence has an effect on the health status over and above the individual effects of deprivation [42]. While various pathways may explain the individual level socio-economic position affecting health, there is a lack of any clear theoretical pathway or mechanism that might link area of residence with health [40,43].

This is the first study from India to investigate the presence of socioeconomic inequalities in dental trauma among adolescents using a relatively affluent group and two groups from extremely deprived strata of the population. It also simultaneously examines the role of various material, psychosocial and behavioural factors in influencing the gradient. The study benefited from a large sample size and achieving a high response rate (86.6%). The scales and questions were adopted from internationally validated questionnaires and in turn were subsequently tested and validated among Indian adolescents.

Despite being standard practice in epidemiological reports in the literature, the use of logistic regression models is not without limitations, particularly in terms of comparing the odds ratios across different models. Adding covariates can increase the variance of the latent variable underlying the dichotomous outcome, and thus odds ratios can increase in magnitude, as was the case when adjusting for material resources in our analyses, when actually the underlying association does not change. And this is the case even if the sample remains the same across models. Therefore, comparisons of the associations across models should be interpreted cautiously, acknowledging that the changes and attenuations may not be exactly as they appear.

There is a need to design cohort studies in order to examine the variations in oral health over a period of time and to investigate the interaction and interplay between various social determinants that influence TDIs. Research to study other psychosocial variables like stress, depression, anxiety and life satisfaction is needed in conjunction with measures of social capital and social support in order to better understand the relative contribution of psychosocial factors towards the socioeconomic gradients in TDIs.

The higher prevalence of trauma in the most deprived areas from our study implies that adolescents in slums and resettlement colonies might need more public health interventions to reduce inequalities in the prevalence of dental trauma. To reduce socioeconomic inequalities in oral health, public health actions should be with a degree and intensity that is proportionate to the level of disadvantage [44]. There is a need to frame policies to reduce exposure of people living in deprived areas to health damaging factors in order to bridge the gap of health inequalities especially in a developing country like India. These results highlight the importance of the physical environment as a very important structural determinant of oral health. Policies aimed at improving the living and working conditions, providing sanitation, supply of safe drinking water and nutrition status might play an instrumental role in reducing inequalities in health in general and oral health in particular.

## Supporting Information

S1 File Dataset for the study. (XLSX)
